# Long-term effects of Cushing’s Disease on visuospatial planning and executive functioning

**DOI:** 10.1192/j.eurpsy.2022.1643

**Published:** 2022-09-01

**Authors:** S. Bauduin, F. Van Haalen, E. Giltay, O. Meijer, A. Pereira, N. Van Der Wee, S. Van Der Werff

**Affiliations:** 1 Leiden University Medical Center, Psychiatry, Leiden, Netherlands; 2 Leiden University Medical Center, Endocrinology, Leiden, Netherlands

**Keywords:** Cushing’s Disease, cognitive planning, executive functioning

## Abstract

**Introduction:**

Patients with remitted Cushing’s Disease (CD) often present persisting impairments in executive and cognitive functioning domains. Little research has been conducted regarding the functional neural correlates of an important executive functioning skill, namely the ability to plan, in these patients.

**Objectives:**

To examine visuospatial planning related brain activity in remitted CD patients and matched controls using functional magnetic resonance imaging (fMRI).

**Methods:**

fMRI scans were made using a 3-Telsa scanner while remitted CD patients (n=21) and age-, gender-, and education matched healthy controls (HCs; n=21) completed a parametric Tower of London (ToL) task. Psychological and cognitive functioning were assessed using validated questionnaires. Clinical severity was assessed retrospectively using the Cushing’s syndrome Severity Index (CSI).

**Results:**

CD Patients were on average 45.1 (SD=7.1) years old, 81% female, and in remission for mean 10.68 (SD=7.69) years. No differences were found in number of correct trials, response times per ToL trial, or in the region of interest analyses. Exploratory wholebrain analyses found that CD patients showed more activation in several brain regions associated with higher cognitive processes on 2-, 3-, and 5-step trials compared to HCs. Over-recruitment of the right parietal operculum cortex in the patients was significantly negatively associated with the prior active disease state on the CSI (r=-0.519, p=0.02).

**Conclusions:**

The increased brain activation during the ToL in remitted CD patients versus controls signals over-recruitment of certain brain areas involved in higher cognitive processes. CD may thus result in long-lasting, subtle scarring effects during demanding executive functioning tasks, despite remission.

**Disclosure:**

No significant relationships.

